# Predicting Adverse Outcomes for Febrile Patients in the Emergency Department Using Sparse Laboratory Data: Development of a Time Adaptive Model

**DOI:** 10.2196/16117

**Published:** 2020-03-26

**Authors:** Sungjoo Lee, Sungjun Hong, Won Chul Cha, Kyunga Kim

**Affiliations:** 1 Department of Digital Health Samsung Advanced Institute for Health Sciences & Technology Sungkyunkwan University Seoul Republic of Korea; 2 Department of Emergency Medicine Samsung Medical Center Sungkyunkwan University School of Medicine Seoul Republic of Korea; 3 Health Information and Strategy Center Samsung Medical Center Seoul Republic of Korea; 4 Statistics and Data Center Research Institute for Future Medicine Samsung Medical Center Seoul Republic of Korea

**Keywords:** order status, sparse laboratory data, time adaptive model, emergency department, adverse outcome, machine learning, imbalanced data

## Abstract

**Background:**

A timely decision in the initial stages for patients with an acute illness is important. However, only a few studies have determined the prognosis of patients based on insufficient laboratory data during the initial stages of treatment.

**Objective:**

This study aimed to develop and validate time adaptive prediction models to predict the severity of illness in the emergency department (ED) using highly sparse laboratory test data (test order status and test results) and a machine learning approach.

**Methods:**

This retrospective study used ED data from a tertiary academic hospital in Seoul, Korea. Two different models were developed based on laboratory test data: order status only (OSO) and order status and results (OSR) models. A binary composite adverse outcome was used, including mortality or hospitalization in the intensive care unit. Both models were evaluated using various performance criteria, including the area under the receiver operating characteristic curve (AUC) and balanced accuracy (BA). Clinical usefulness was examined by determining the positive likelihood ratio (PLR) and negative likelihood ratio (NLR).

**Results:**

Of 9491 eligible patients in the ED (mean age, 55.2 years, SD 17.7 years; 4839/9491, 51.0% women), the model development cohort and validation cohort included 6645 and 2846 patients, respectively. The OSR model generally exhibited better performance (AUC=0.88, BA=0.81) than the OSO model (AUC=0.80, BA=0.74). The OSR model was more informative than the OSO model to predict patients at low or high risk of adverse outcomes (*P*<.001 for differences in both PLR and NLR).

**Conclusions:**

Early-stage adverse outcomes for febrile patients could be predicted using machine learning models of highly sparse data including test order status and laboratory test results. This prediction tool could help medical professionals who are simultaneously treating the same patient share information, lead dynamic communication, and consequently prevent medical errors.

## Introduction

For time-sensitive diseases, timely decisions are essential; however, the availability of data is extremely limited in the early stages of medicine [1,2]. Data obtained in the long term after the patient’s visit provide sufficient information, and the results of analysis to predict the patient’s outcome are highly accurate. However, the timing and effectiveness of this long-term data are limited in early decision making because the results do not reflect the patient’s initial status. Therefore, it is necessary to develop a time adaptive model that reflects the decision-making process by utilizing the pattern of interim information in uncertain situations during the initial stages of the patient’s visit.

Biomarkers, especially those obtained via laboratory data, play a key role in clinical decisions in emergency settings [3-5]. Laboratory data are important for predicting the patient’s prognosis but can lead to delays in decision making since many test results are not available during the initial stages [6-9]. This further exacerbates the inherent problems with laboratory data including a high level of sparsity due to the many test types and variation in individual orders [8,10]. Therefore, we try to maximize the utilization of laboratory information through patterns and the use of order status, which can infer the patient’s initial status before obtaining test results.

Previous studies have focused on utilizing a sufficient amount of laboratory test data. Most predictive models have been developed based on long intervals such as those to predict mortality occurring within 24 or 48 hours rather than earlier periods; with these longer periods, researchers can be guaranteed of adequate information from test results [11,12]. Previous studies have also used a limited number of the frequently measured test variables, rather than including all possible tests, to predict mortality, and they found that sufficient data were available to develop prediction models [13,14].

This study aimed to develop time adaptive models that predict adverse outcomes for febrile patients in the emergency department (ED) based on a machine learning approach and highly sparse data.

## Methods

### Study Setting

This retrospective study was conducted with ED data from a tertiary academic hospital in Seoul, Korea. The hospital has approximately 2000 beds. The outpatient department has an average of approximately 9000 patients per day, while the ED has approximately 220 patients per day. Since the opening of its comprehensive cancer center in 2003, the hospital has a large portion of oncology patients undergoing both surgical and medical procedures. This study was approved by the institutional review board of the study site (IRB File No: SMC 2018-08-125). This report follows the Transparent Reporting of a Multivariable Prediction Model for Individual Prognosis or Diagnosis (TRIPOD) reporting guideline.

### Source of Data

Data were obtained from a clinical data warehouse containing medical data for research, which enables de-identification and retrieval of patient information from electronic medical records for research purposes. It uses global standard terminology and provides near realtime data through daily updates. In addition to basic patient demographics, it provides information on tests, medications, diagnoses, and operations.

### Participants

Patients who visited the ED from March 2017 through February 2019 were included in the study. Then, only febrile (body temperature >38°C) [15] and adult patients (aged ≥18 years) were included. Patients were excluded if the main reason for their visit was determined as trauma.

### Outcome and Predictors

We used a binary composite outcome for severity. Severity was considered as death or admission to the intensive care unit after transfer from the ED.

**Figure 1:**
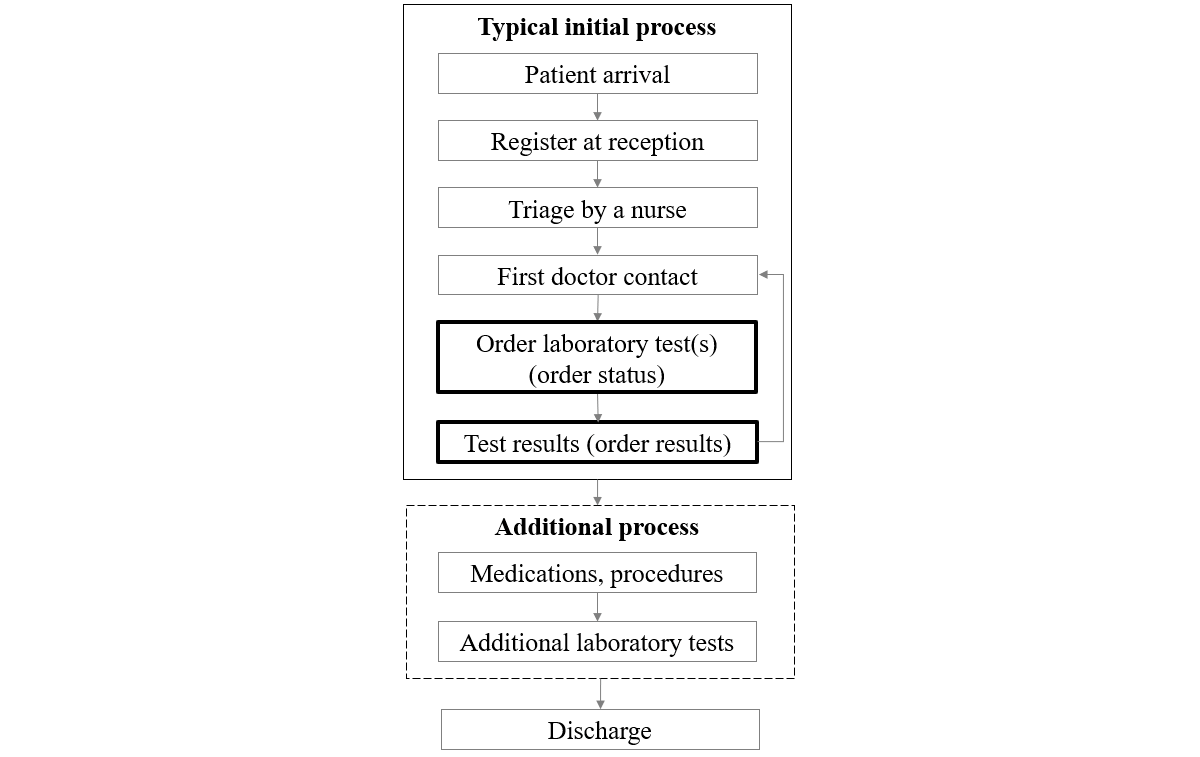
Process flow in the emergency department.

Only laboratory test data were used as predictors, and the list of laboratory tests was selected by experts. Predictors were selected based on the typical ED process in which all possible laboratory tests could be performed after the initial assessment by physicians [16]. Figure 1 shows the general process from the typical initial process to patient discharge from the ED. This study focused on the initial process and the ordering of laboratory tests and test results in particular. Two models were developed from the viewpoint of the initial process, using laboratory test order status and laboratory test results that became available later.

**Figure 2:**
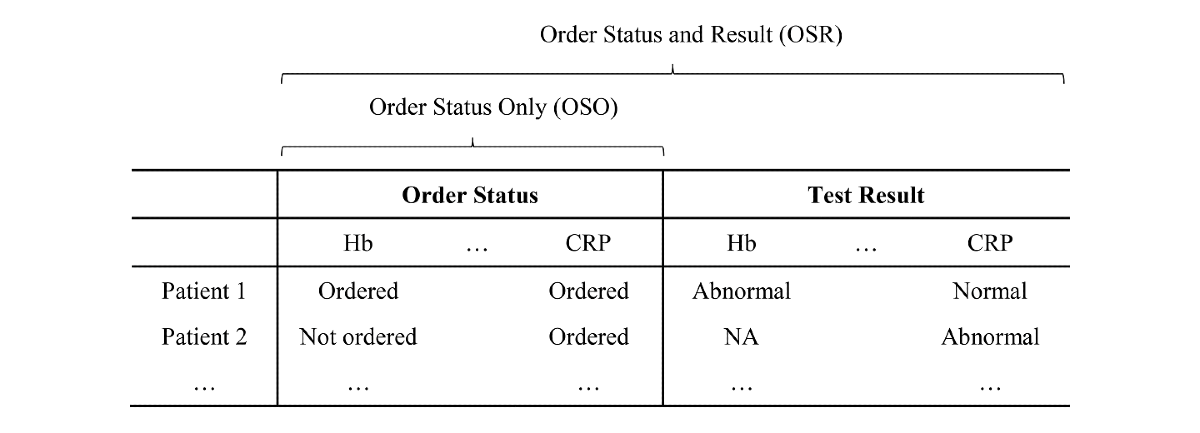
Representative example of the range of predictors for each model, where each row indicates a patient’s record of laboratory tests. Additional laboratory tests and patient records can be added. Order status, which indicates whether the test was ordered, was used in the OSO model. The OSR model was developed using order status and test results, which had three levels: normal, abnormal, and NA (not reported). CRP: C-reactive Protein; Hb: hemoglobin.

The laboratory test data provide the order status and result for each laboratory test, and all the variables were categorized. Order status indicates whether a patient has an order for a laboratory test, and the test result reflects whether it was normal, abnormal, or not reported. When the test was repeatedly performed, only the first test data were included. We developed two predictive models using these laboratory test data: order status only (OSO) for the first model and order status and results (OSR) for model 2 (Figure 2).

For a group of laboratory tests that are not frequently ordered but are conducted for only a few patients, the order status information causes severe data sparsity. Rather than using the order status information for each of those tests, new variables were introduced. First, rarely ordered tests (ROTs) were identified as tests that had an ordered rate <5%. The new variable ROT was defined as the number of ROTs ordered for each patient. Likewise, for tests that generate rarely detected abnormal results (RARs; <5% of the results are abnormal), a new variable RAR was defined as the number of RARs obtained among those tests for each patient.

### Analytical Methods

Patients were randomly assigned to two cohorts for model development (70%) and validation (30%), which had similar distributions with respect to the outcome. We applied and compared various machine learning methods, including random forest (RF), support vector machine, logistic regression with least absolute shrinkage and selection operator, ridge, and elastic net (EN) regularization [17-21]. For optimization, the grid search was used for all algorithms, and the stochastic gradient descent method was used for penalized regression algorithms. The Gini index was selected to measure the split quality in RF. Linear and sigmoid kernels were considered for the support vector machine. Moreover, hyperparameters for each algorithm were tuned with the accuracy measure based on 10-fold cross-validations that were repeatedly conducted 5 times to reduce the partition bias in model development.

The predictive models were evaluated with the validation cohort using various performance measures, such as the area under the receiver operating characteristic curve (AUC), area under the precision recall curve (AUPRC), balanced accuracy (BA), sensitivity, specificity, *F*1 score, positive likelihood ratio (PLR), and negative likelihood ratio (NLR) [22]. For each measure, we provide 95% CIs, which were estimated by bootstrapping 2000 resamples, and selected the optimal thresholds with which BA was maximized. Moreover, net reclassification improvement [23] was used to measure the incremental value of adding test results to the order status in the prediction. Comparison of the performance between the models was conducted using the bootstrap-t method [24]. The resulting predictive models were further compared with the Modified Early Warning Score (MEWS), a reference algorithm currently used to predict the severity of a patient’s condition in clinical practice [25]. The clinical usefulness of the models was demonstrated in two ways. First, we conducted Spearman correlation analysis to evaluate whether clinically meaningful variables were selected consistently across the algorithms. Second, the Kaplan-Meier method and log-rank test were used to estimate and compare survival curves between high-risk and low-risk groups as predicted from the OSR model. We used 2-sided *P*<.05 for statistical significance.

Class imbalance existed in our outcome data. This can lead to the classifier having poor performance because it can create bias against a class and may not able to distinguish between noise and the individuals from the minority class [26,27]. We investigated its effects on prediction performance using various scenarios in which different techniques and class ratio were considered for imbalance reduction. Three oversampling and three undersampling approaches were considered. The oversampling methods included random, synthetic minority oversampling technique, and adaptive synthetic sampling. The undersampling methods included random, NearMiss-2, and edited nearest neighbors [28]. Furthermore, we increased the ratio between the minority and majority classes from 1:1 to 1:10 and tried to find the best performance. For each scenario, we used EN for model development, and the performance was assessed using 100 bootstrap resamples from the original dataset.

The preprocess was conducted using R version 3.4.4 [29], and the analytic process was performed with Python version 3.6.2 (Python Software Foundation, Wilmington, DE) using pandas, numpy, sklearn, and imblearn library. More details related to the model development are available in Multimedia Appendix 1.

## Results

### Patient Demographics

A total of 154,402 patients visited the ED between March 1, 2017, and February 28, 2019. Based on the inclusion and exclusion criteria, 9491 patients remained in the final dataset used for modeling (Multimedia Appendix 2). The randomly divided model development and validation cohorts included 6645 and 2846 patients, respectively, with a composite adverse outcome frequency of 4.6% in the 2 datasets. The baseline characteristics reflect only the initial patient status. The mean age (SD) was 55.2 years (17.7 years); 4839 of the 9491 patients (51.0%) were female; and 432 of the 9491 patients (4.6%) experienced the composite adverse outcome. Patients in the development and validation cohorts were similarly distributed (Table 1).

The three most frequently observed laboratory tests were C reactive protein, chlorine, and sodium. Among a total of 286 laboratory tests after preprocessing, 201 ROTs (order rate <5%) and 231 tests with RARs (abnormal rate <5%) were identified. The OSO model had 85 order status variables as well as the ROT variable. Similarly, the OSR model had 55 result variables, the RAR variable, and the variables in the OSO model.

**Table 1:** Baseline characteristics of the total sample and comparisons between the two patient cohorts used to develop and validate the two models.

Characteristic		Total sample	Model development cohort (n=6645)	Model validation cohort (n=2846)	*P* value
**Sex, n (%)**					
	Female	4839 (51.0)	3399 (51.2)	1440 (50.6)	.64
	Male	4652 (49.0)	3246 (48.8)	1406 (49.4)	
Age (years), mean (SD)		55.2 (17.7)	55.0 (17.8)	55.6 (17.6)	.17
**Transportation, n (%)**					
	Other	7483 (78.8)	5224 (78.6)	2259 (79.4)	.42
	Ambulance	2008 (21.2)	1421 (21.4)	587 (20.6)	
**Route, n (%)**					
	Indirect	1217 (12.8)	830 (12.5)	387 (13.6)	.15
	Direct	8274 (87.2)	5815 (87.5)	2459 (86.4)	
**Mentality, n (%)**					
	Alert	9206 (97.0)	6449 (97.1)	2757 (96.9)	.69
	Not alert	285 (3.0)	196 (2.9)	89 (3.1)	
**Pulse rate, n (%)**					
	Normal (60-120 beats per minute)	7108 (75.1)	4956 (74.8)	2152 (75.7)	.35
	Abnormal (<60 or >120 beats per minute)	2361 (24.9)	1671 (25.2)	690 (24.3)	
**Respiratory rate, n (%)**					
	Normal (10-30 breaths per minute)	9399 (99.2)	6577 (99.2)	2822 (99.2)	1.00
	Abnormal (<10 or >30 breaths per minute)	73 (0.8)	51 (0.8)	22 (0.8)	
**Systolic blood pressure, n (%)**					
	Normal (90-140 mmHg)	7212 (76.0)	5044 (75.9)	2168 (76.2)	.80
	Abnormal (<90 or >140 mmHg)	2279 (24.0)	1601 (24.1)	678 (23.8)	
**Diastolic blood pressure, n (%)**					
	Normal (60-90 mmHg)	6790 (71.5)	4738 (71.3)	2052 (72.1)	.44
	Abnormal (<60 or >90 mmHg)	2701 (28.5)	1907 (28.7)	794 (27.9)	
**SpO_2_^a^, n (%)**					
	Normal (>90)	9070 (97.4)	6356 (97.5)	2714 (97.2)	.48
	Abnormal (<90)	245 (2.6)	166 (2.5)	79 (2.8)	
**Outcome, n (%)**					
	Normal	9059 (95.4)	6342 (95.4)	2717 (95.5)	.99
	Composite adverse outcome^b^	432 (4.6)	303 (4.6)	129 (4.5)	

^a^SpO_2_ : peripheral oxygen saturation.

^b^Defined as death or admission to the intensive care unit.

### Model Performance and Specification

The OSO and OSR models were each developed based on 5 different algorithms. The RF-based models were selected as the final predictive OSO and OSR models because they had better performance overall in terms of the most evaluation measures, including specificity, precision, *F*1 score, NLR, and PLR (Multimedia Appendix 3). Note that the EN-based OSR model was comparable to the RF-based OSR model. Compared to the MEWS (AUC = 0.68), the final OSO and OSR models showed significant AUC improvement, at 12% and 20%, respectively (Table 2, Multimedia Appendix 4). Both models had better performance than the MEWS according to most of the other measures, including the AUPRC, maximum BA, and *F*1 score.

**Table 2:** Internal validation of the models using different laboratory information, reported as the score and 95% CI.

Measure^a^	MEWS^b^	OSO^c^	OSR^d^	Difference (MEWS vs OSO)^e^	Difference (OSO vs OSR)^f^
AUC^g^	0.68 (0.63 to 0.73)	0.80 (0.76 to 0.84)	0.88 (0.85 to 0.91)	0.12 (0.12 to 0.12)	0.08 (0.08 to 0.08)
AUPRC^h^	0.14 (0.10 to 0.20)	0.25 (0.18 to 0.33)	0.39 (0.30 to 0.47)	0.11 (0.11 to 0.11)	0.14 (0.14 to 0.14)
Sensitivity	0.49 (0.42 to 0.61)	0.70 (0.62 to 0.82)	0.81 (0.76 to 0.89)	0.22 (0.21 to 0.22)	0.10 (0.10 to 0.10)
Specificity	0.82 (0.66 to 0.83)	0.78 (0.66 to 0.83)	0.81 (0.75 to 0.83)	–0.04 (–0.04 to –0.04)	0.04 (0.04 to 0.04)
Balanced accuracy	0.65 (0.62 to 0.69)	0.74 (0.71 to 0.77)	0.81 (0.78 to 0.84)	0.09 (0.09 to 0.09)	0.07 (0.07 to 0.07)
Precision	0.11 (0.08 to 0.14)	0.13 (0.10 to 0.16)	0.17 (0.13 to 0.20)	0.02 (0.02 to 0.02)	0.04 (0.04 to 0.04)
*F*1 score	0.18 (0.14 to 0.22)	0.22 (0.17 to 0.26)	0.28 (0.23 to 0.32)	0.04 (0.04 to 0.04)	0.06 (0.06 to 0.06)
PLR^i^	2.68 (1.76 to 3.27)	3.10 (2.25 to 4.29)	4.22 (2.92 to 4.94)	0.49 (0.48 to 0.5)	1.07 (1.06 to 1.08)
NLR^j^	0.63 (0.49 to 0.73)	0.39 (0.24 to 0.49)	0.23 (0.12 to 0.31)	–0.25 (–0.25 to –0.25)	–0.14 (–0.15 to –0.14)

^a^Calculations were completed with the validation set, and 95% CIs were computed using 2000 bootstrap replicates for each performance measure.

^b^MEWS: Modified Early Warning Score.

^c^OSO: model with order status only.

^d^OSR: model with both order status and test result.

^e^Difference in each performance measure between the MEWS and OSO model.

^f^Difference in each performance measure between the OSO and OSR models.

^g^AUC: area under the receiver operating characteristic curve.

^h^AUPRC: area under the precision recall curve.

^i^PLR: positive likelihood ratio.

^j^NLR: negative likelihood ratio.

**Figure 3:**
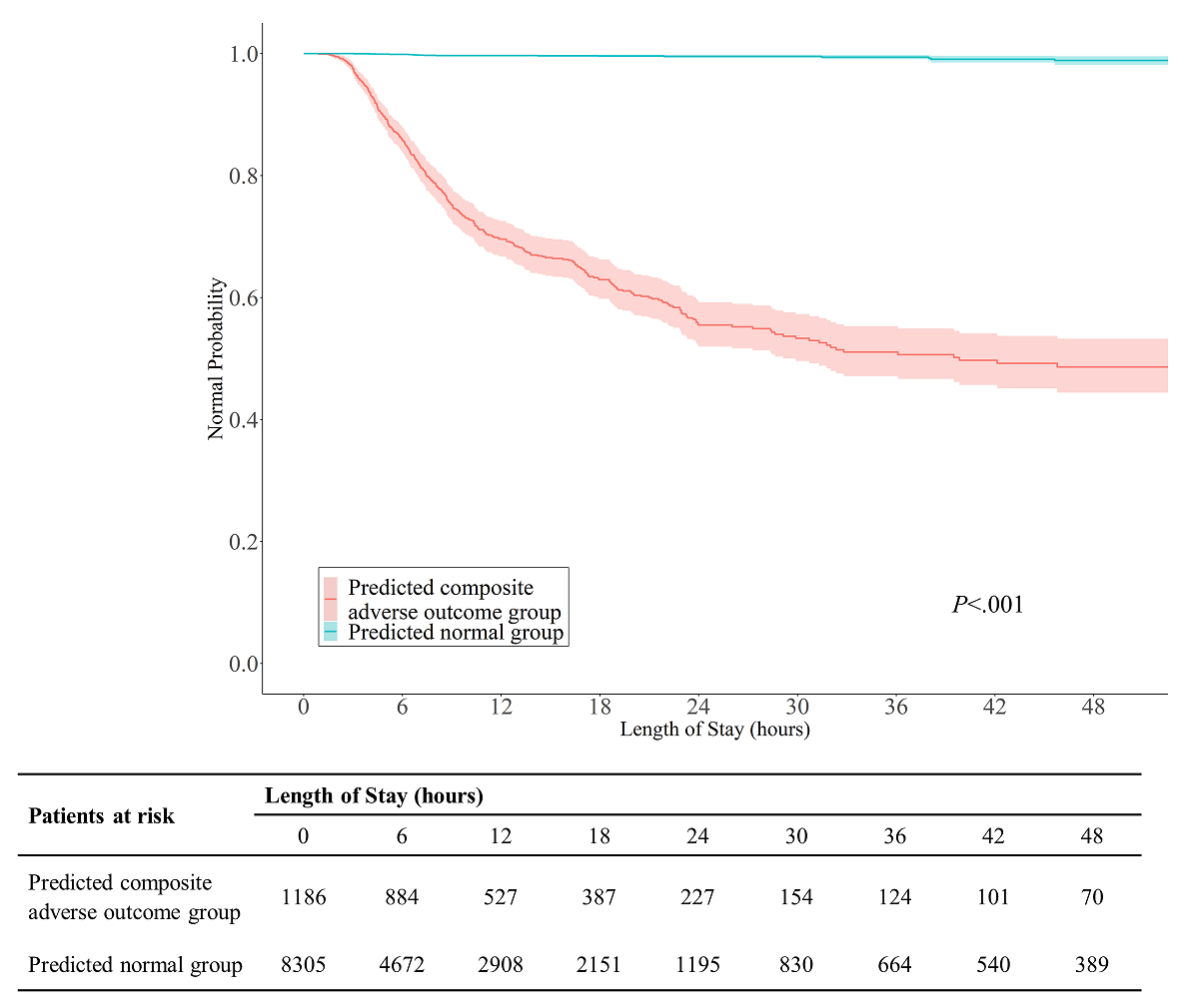
The curves indicate how the actual outcome developed over time when the patients were divided into high-risk and low-risk categories, as predicted from the OSR model. The graph was plotted using the Kaplan-Meier survival curve, and the P-value shows the log-rank test result.

Compared with the OSO model, the OSR model showed significant improvement in the AUC, at 8%, and maximum BA, at 7%. Additionally, the OSR model was more informative than the OSO model in predicting low-risk and high-risk patients in terms of outcome (*P*<.001 for difference in both PLR and NLR). A significant additional increment in reclassification was also observed between the OSO and OSR models (net reclassification improvement=0.15). Despite the lack of information from laboratory test results, the OSO model showed considerable performance (AUC = 0.80). Therefore, the order pattern itself can be important information for prediction, and it is better to use the OSO model in the early stages than to wait until test results are obtained. However, because it utilizes laboratory test results obtained later in time, the OSR model has higher accuracy and better performance than the OSO model. According to the Kaplan-Meier survival curves (log-rank test, *P*<.001) comparing the predicted outcome groups, the OSR model can classify patients well (Figure 3). The complementary use of these two models can be beneficial both before and after all laboratory test results are available in clinical situations such as those in the ED.

Important variables selected from the RF-based and EN-based models were moderately correlated in terms of their value importance and odds ratios, respectively (*r*_s_=0.603 and 0.626 for the OSO and OSR models, respectively; Multimedia Appendix 5). Among the top 10 variables selected in each of the RF-based and EN-based models, 80% and 60%, respectively, were shared by the OSO and OSR models. Therefore, the important variables were very similar between the RF and EN models, potentially suggesting our models are robust, regardless of the algorithm used. The order statuses of cardiac troponin I, creatine kinase, and creatine kinase-MB were the top 3 variables in terms of importance in both the RF-based and EN-based OSO models. The order status of creatine kinase remained in the top 10 important variables in the OSR models. The lactic acid test result was the most important variable in both the RF-based and EN-based OSR models.

The data had severe outcome imbalance: 95.4% (9059) for the majority class and 4.6% (432) for the minority class. However, the sensitivity analysis to calibrate the imbalance with various reduction scenarios did not reveal any considerable improvement in the prediction performance. Therefore, our models are not affected much by the imbalance problem (Multimedia Appendix 6).

## Discussion

### Principal Findings

In this study, we developed a time adaptive model to predict adverse outcomes for patients in the ED. These patients are likely to have insufficient and unconfirmed clinical information, especially in the early stages of the ED process. The OSO model, which only utilizes test order status, supports our hypothesis that it is feasible to predict patient prognosis based only on the fact that a laboratory test has been ordered and without the test results. Patient demographics or vital signs were also not required for the prediction.

Febrile patients have a considerable number of laboratory tests to consider. The ED receives patients with different illnesses and febrile patients with various diseases in particular. Fever is also the most common sign of potential sepsis [30,31], requiring more laboratory tests. Febrile patients were selected as the target population to investigate the sparsity and large number of tests typically required initially and to reflect as many tests as possible.

The OSO model mimics the ED physician’s clinical reasoning process in practical settings, while prediction models developed previously are limited by using only confirmed results [11-14]. For time-sensitive conditions, multiple tests are performed simultaneously, resulting in a combination of confirmed and unconfirmed results, which necessitates models that can be applied in real practice. This study is the first step to overcome these limitations. Furthermore, it is possible to predict the initial outcome of patients with a severe condition using the OSO model and then update the prediction using the OSR model when additional information becomes available in a time adaptive manner.

In modern medicine, a multidisciplinary approach is a cornerstone of better quality [32,33]. In the ED, multiple providers work as a team to simultaneously treat various conditions [7]. Analyzing laboratory test orders and results could benefit the whole treatment team by interpreting the initial impression and intention of physicians who give the orders for tests. It is possible that each physician may not be aware of the intention of others while simultaneously treating the same patient [7,34,35]. In this context, the prediction tool can help share information, lead dynamic communication, and consequently prevent medical errors.

This study could be expanded further by including vital signs, procedures, and medications for better prediction. In addition, the application could be broadened to include diagnosis as well as adverse outcomes, especially for diseases where the patient’s response over time after a particular treatment is important. Additionally, it can be extended to anticipate clinical decisions, which may be integrated as clinical decision support. The time variable is the most essential component for these predictions, and this model has successfully shown its feasibility.

### Limitations

This study has some limitations. First, the models were developed and internally validated using data from a single large hospital. Although cross-validation was performed with repetition, optimization, and several candidates of hyperparameters, along with survival analysis to increase their clinical impact, further studies are required for external and prospective validation.

Second, the primary parameters such as laboratory results, which may vary across individuals and clinical fields, were from febrile patients. Therefore, it could be difficult to apply these to other populations, although we attempted to include as many tests as possible. However, we believe the important variables that were selected related to laboratory tests from the 2 models are clinically relevant for the outcome variables, so there is potential to extend the models to other target populations in future studies.

Third, the OSO and OSR models were not developed with a continuous time sequence. Instead of creating a continuous model, we tried to build representative models to reflect test order status and results. Further research will be required to create a continuous model for practical use, which can be applied to various time thresholds.

Last, the imbalance of data could have affected the performance of models developed using raw data. Although various methods to deal with the issues related with imbalanced data were applied to develop and validate the model, only a few of algorithms among the methods for calibrating the class imbalance were used. It is possible that the use of other algorithms would have changed the results even though the results in this study were not significantly improved after addressing the issue of imbalanced data. Therefore, various additional algorithms should be used to address the imbalanced data in future studies.

### Conclusions

Adverse outcomes during the early stages for febrile patients could be predicted using a time adaptive model and machine learning approach based on the highly sparse data from test order status and laboratory test results.

## Appendix

Multimedia Appendix 1 Supplemental code for developing models (OSO).

Multimedia Appendix 2 Number of laboratory log per person.

Multimedia Appendix 3 Performance of the OSO and OSR models.

Multimedia Appendix 4 Receiver operating characteristic (ROC) curves for models.

Multimedia Appendix 5 Important variables selected from developed models.

Multimedia Appendix 6 Model performance without and with applying imbalance-easing techniques.

## Abbreviations

AUC: area under the receiver operating characteristic curve.
AUPRC: area under the precision recall curve.
BA: balanced accuracy.
CRP: C reactive protein.
ED: emergency department.
EN: elastic net.
Hb: hemoglobin.
MEWS: Modified Early Warning Score.
NLR: negative likelihood ratio.
OSO: order status only.
OSR: order status and result.
PLR: positive likelihood ratio.
RAR: number of rarely detected results.
RF: random forest.
ROT: number of rarely ordered tests.
TRIPOD: Transparent Reporting of a Multivariable Prediction Model for Individual Prognosis or Diagnosis.
