# Supplementary material for: Predicting Adverse Outcomes for Febrile Patients in the Emergency Department Using Sparse Laboratory Data: Development of a Time Adaptive Model
Source: JMIR Med Inform. 2020 Mar 26;8(3):e16117. doi: 10.2196/16117 (PMC7146241; doi:10.2196/16117)
Supplement: Multimedia Appendix 1 [file medinform_v8i3e16117_app1.pdf]

Multimedia Appendix 1. Supplemental code for developing models (OSO)

[1]:
```
# ================================================
#  The version of library
#  - scikit-learn (0.20.3)
#  - imbalanced-learn (0.5.0)
# ================================================

import pandas as pd
import numpy as np
from sklearn.linear_model import Lasso, SGDClassifier
from sklearn.model_selection import KFold,  RepeatedKFold, GridSearchCV,␣
 ↪RandomizedSearchCV, cross_val_score
from sklearn.svm import SVC
from sklearn.ensemble import RandomForestClassifier
```

1 Model development

[ ]:
```
# ================================================
# 1. lasso
# ================================================
tuned_parameters = [{'alpha':  np.logspace(-15, 0.5, 80)}]
cv = RepeatedKFold(n_splits = 10, n_repeats = 5, random_state = 3033)
grid_lasso = GridSearchCV(SGDClassifier(loss='log', penalty='l1', l1_ratio=␣
 ↪1,random_state = 3033)
                          ,tuned_parameters, cv=cv, scoring = 'accuracy',␣
 ↪return_train_score = True)
grid_lasso.fit(X_train1, y_train1)
```

[ ]:
```
# ================================================
# 2. Ridge
# ================================================
tuned_parameters = [{'alpha':  np.logspace(-15, 0.5, 80)}]
cv = RepeatedKFold(n_splits=10, n_repeats=5, random_state = 3033)
```

```
grid_ridge = GridSearchCV(SGDClassifier(loss='log', penalty='l2', l1_ratio= 0,␣
 ↪random_state = 3033)
                          ,tuned_parameters, cv=cv, scoring = 'accuracy',␣
 ↪return_train_score = True)
grid_ridge.fit(X_train1, y_train1)
```

[ ]:
```
# ===============================================
# 3. elastic net
# ===============================================
tuned_parameters = [{'alpha':  np.logspace(-15, 0.5, 80)}]
cv = RepeatedKFold(n_splits=10, n_repeats=5, random_state = 3033)

forEN = np.arange(0.1,1.0,0.2)
for i in range(0,5):
    grid_EN = GridSearchCV(SGDClassifier(loss='log',␣
 ↪penalty='elasticnet',l1_ratio=forEN[i],random_state = 3033)
                          ,tuned_parameters, cv=cv, scoring = 'accuracy',␣
 ↪return_train_score = True)
    grid_EN.fit(X_train1, y_train1)
```

[ ]:
```
# ===============================================
# 5. RandomForest -grid, 516
# ===============================================
cv = RepeatedKFold(n_splits=10, n_repeats=5, random_state = 3033)
tuned_parameters = [{'max_features': np.arange(5,16),
                     'max_depth' :[10, 20, 30, 40, 50, 60, 70, 80, 90, 100,␣
 ↪None],
                     'min_samples_leaf': [1, 2, 5, 10, 15, 20],
                     'min_samples_split': [2, 5, 10, 15]}]

grid_rf = GridSearchCV(RandomForestClassifier(n_estimators=1000, bootstrap =␣
 ↪True, random_state=3033),
                       tuned_parameters, cv=cv, scoring = 'accuracy',␣
 ↪return_train_score = True)
grid_rf.fit(X_train1, y_train1)
```

[ ]:
```
# ===============================================
# 6. SVM
# ===============================================
cv = RepeatedKFold(n_splits=10, n_repeats=5, random_state = 3033)

tuned_parameters_sigmoid = [{'kernel': ['sigmoid'], 'gamma': 2.0**-np.
 ↪arange(-15,3),
                             'C': 2.0**-np.arange(-5,15)}]
tuned_parameters_rbf = [{'kernel': ['rbf'], 'gamma': 2.0**-np.arange(-15,3),
                             'C': 2.0**-np.arange(-5,15)}]
```

```
tuned_parameters_linear = [{'kernel': ['linear'], 'C': 2.0**-np.arange(-5,15)}]

tune = {"sigmoid": tuned_parameters_sigmoid, "rbf": tuned_parameters_rbf,␣
↪"linear": tuned_parameters_linear}
tune_name = ["sigmoid", "rbf", "linear"]

for i in range(3):
    grid_svm = GridSearchCV(SVC(random_state = 3033, probability = True),
                            tune[tune_name[i]], cv=cv, scoring = 'accuracy',␣
↪return_train_score = True)
    grid_svm.fit(X_train1, y_train1)
```

2 Imbalance

2.1 Undersampling

[ ]:
```
from collections import Counter
from imblearn.datasets import make_imbalance
from imblearn.under_sampling import RandomUnderSampler, NearMiss,␣
↪EditedNearestNeighbours
```

[ ]:
```
# ===============================================
# original dataset
# ===============================================
print('Original dataset shape for training {}'.format(Counter(y_train1)))
print('Original dataset shape for testing {}'.format(Counter(y_test1)))
```

[ ]:
```
# ===============================================
# Making function_ evaluation_ Elasticnet
# ===============================================
def under_imb_en(imb_method, X_train, y_train):

    X_bal_ = {}; y_bal_ = {}
    grid_search_ = {}; final_model_ = {}
    lambda1_ = [None]*10; auc_under_ = [None]*10; auprc1_ = [None]*10

    for i in range(0,10):

        # balance process
        if imb_method == RandomUnderSampler:
            rus = imb_method(sampling_strategy= 1/(i+1), random_state = 3033)
        elif imb_method == NearMiss:
            rus = imb_method(sampling_strategy = 1/(i+1), random_state = 3033,␣
↪version = 2)
        elif imb_method == EditedNearestNeighbours:
```

```
            rus = imb_method(sampling_strategy ='majority', random_state =␣
↪3033, n_neighbors = neighbors_list[i])

        warnings.simplefilter('ignore')
        X_res, y_res = rus.fit_sample(X_train, y_train)
        print('\n')
        print('sampling_algorithm : {} \n balanced sampling_strategy={}'
              .format(imb_method.__name__, Counter(y_res)))

        X_bal = pd.DataFrame(X_res); X_bal.columns = list(X_train)
        y_bal = pd.DataFrame(y_res)
        print("X_under shape: ", X_bal.shape)
        print("y_under shape: ", y_bal.shape)

        # parameter tuning process
        tuned_parameters = [{'alpha': np.logspace(-15, 0.5, 80)}]
        cv = RepeatedKFold(n_splits=10, n_repeats=5, random_state = 3033)
        grid_search = GridSearchCV(SGDClassifier(loss='log',␣
↪penalty='elasticnet',
                                                l1_ratio=0.5, random_state =␣
↪3033),
                                  tuned_parameters, cv=cv, scoring =␣
↪'accuracy', return_train_score = True)

        grid_search.fit(X_bal, y_bal)
        lambda1 = grid_search.best_params_["alpha"]

        # final model fitting for elasticnet
        final_model = SGDClassifier(loss='log', penalty = 'elasticnet',␣
↪shuffle=True,
                                verbose=False, average=False,random_state =␣
↪3033, l1_ratio = 0.5)
        final_model.set_params(alpha=grid_search.best_params_["alpha"])

        print("Alpha of CV = ", grid_search.best_params_)
        print("Alpha of final=", final_model.get_params)

        final_model.fit(X_bal, y_bal)

        # AUC
        y_pred_prob = final_model.predict_proba(X_test1.values)
        fpr_d, tpr_d, _ = roc_curve(y_test1, y_pred_prob[:,1])
        auc_under= auc(fpr_d, tpr_d)
        print('AUC for undersampling = ', round(auc_under,4))

        # AUPRC
```

```
        precision_auprc, recall_auprc, thresholds_auprc =␣
↪precision_recall_curve(y_test1, y_pred_prob[:, 1])
        auprc1 = auc(recall_auprc, precision_auprc); print("AUPRC for␣
↪undersampling = ", round(auprc1,4))

        X_bal_[i] = X_bal; y_bal_[i] = y_bal;
        grid_search_[i] = grid_search; lambda1_[i] = lambda1
        final_model_[i] = final_model;
        auc_under_[i] = auc_under; auprc1_[i] = auprc1

    # save the result of evaluation as dataframe type
    Result_imb = {'X_bal' : X_bal_, 'y_bal' : y_bal_,
                  'grid_search' : grid_search_, 'final_model' : final_model_,
                  'lambda' : lambda1_, 'AUC': auc_under_, 'AUPRC' : auprc1_}

    pickle.dump(Result_imb, open("" + filename + ".pkl", 'wb'))

    return(Result_imb)
```

[ ]:
```
neighbors_list = [246, 155, 152, 135, 84, 59, 47, 40, 33,28]
under_imb_en(EditedNearestNeighbours, X_train1, y_train1)
under_imb_en(RandomUnderSampler, X_train1, y_train1)
under_imb_en(NearMiss, X_train1, y_train1)
```

2.2 Oversampling

[ ]:
```
from collections import Counter
from imblearn.datasets import make_imbalance
from imblearn import over_sampling
from imblearn.over_sampling import RandomOverSampler, SMOTE, ADASYN
```

[ ]:
```
# ================================================
# Making function_ evaluation_ Elasticnet
# ================================================
def over_imb_en(imb_method, X_train, y_train):

    X_bal_ = {}; y_bal_ = {}
    grid_search_ = {}; final_model_ = {}
    lambda1_ = [None]*10; auc_under_ = [None]*10; auprc1_ = [None]*10

    for i in range(0,10):
        sampling_ratio = {1:  minority[i], 0: majority[i]}
        # balance process
        if imb_method == RandomOverSampler:
            rus = imb_method(sampling_strategy = sampling_ratio, random_state =␣
↪3033)
```

```
        elif imb_method == SMOTE:
            rus = imb_method(sampling_strategy = sampling_ratio,␣
↪random_state=3033)
        elif imb_method == ADASYN:
            rus = imb_method(sampling_strategy = sampling_ratio, random_state =␣
↪3033)

        warnings.simplefilter('ignore')
        X_res, y_res = rus.fit_sample(X_train, y_train)
        print('\n')
        print('sampling_algorithm : {} \n balanced sampling_strategy={}'
              .format(imb_method.__name__, Counter(y_res)))

        X_bal = pd.DataFrame(X_res); X_bal.columns = list(X_train)
        y_bal = pd.DataFrame(y_res)
        print("X_under shape: ", X_bal.shape)
        print("y_under shape: ", y_bal.shape)

        # parameter tuning process
        tuned_parameters = [{'alpha': np.logspace(-15, 0.5, 80)}]
        cv = RepeatedKFold(n_splits=10, n_repeats=5, random_state = 3033)
        grid_search = GridSearchCV(SGDClassifier(loss='log',␣
↪penalty='elasticnet',
                                          l1_ratio=0.5, random_state =␣
↪3033),
                                  tuned_parameters, cv=cv, scoring =␣
↪'accuracy', return_train_score = True)

        grid_search.fit(X_bal, y_bal)
        lambda1 = grid_search.best_params_["alpha"]

        # final model fitting for elasticnet
        final_model = SGDClassifier(loss='log', penalty = 'elasticnet',␣
↪shuffle=True,
                                verbose=False, average=False,random_state =␣
↪3033, l1_ratio = 0.5)
        final_model.set_params(alpha=grid_search.best_params_["alpha"])

        print("Alpha of CV = ", grid_search.best_params_)
        print("Alpha of final=", final_model.get_params)

        final_model.fit(X_bal, y_bal)

        # AUC
        y_pred_prob = final_model.predict_proba(X_test1.values)
        fpr_d, tpr_d, _ = roc_curve(y_test1, y_pred_prob[:,1])
```

```
        auc_under= auc(fpr_d, tpr_d)
        print('AUC for undersampling = ', round(auc_under,4))

        # AUPRC
        precision_auprc, recall_auprc, thresholds_auprc =␣
↪precision_recall_curve(y_test1, y_pred_prob[:, 1])
        auprc1 = auc(recall_auprc, precision_auprc); print("AUPRC for␣
↪undersampling = ", round(auprc1,4))

        X_bal_[i] = X_bal; y_bal_[i] = y_bal;
        grid_search_[i] = grid_search; lambda1_[i] = lambda1
        final_model_[i] = final_model;
        auc_under_[i] = auc_under; auprc1_[i] = auprc1

    # dataframe    evaluation
    Result_imb = {'X_bal' : X_bal_, 'y_bal' : y_bal_,
                  'grid_search' : grid_search_, 'final_model' : final_model_,
                  'lambda' : lambda1_, 'AUC': auc_under_, 'AUPRC' : auprc1_}

    return(Result_imb)
```

[ ]:
```
over_imb_en(RandomOverSampler, X_train1, y_train1)
over_imb_en(SMOTE, X_train1, y_train1)
over_imb_en(ADASYN, X_train1, y_train1)
```
