# Supplementary material for: Predicting Adverse Outcomes for Febrile Patients in the Emergency Department Using Sparse Laboratory Data: Development of a Time Adaptive Model
Source: JMIR Med Inform. 2020 Mar 26;8(3):e16117. doi: 10.2196/16117 (PMC7146241; doi:10.2196/16117)
Supplement: Multimedia Appendix 3 [file medinform_v8i3e16117_app3.pdf]

Multimedia Appendix 3. Performance of the OSO and OSR models.

OSO					
**Measure[a]**	**RandomForest**	**LASSO**	**Elastic Net**	**Ridge**	**SVM**
AUC[b]	0.80 (0.76, 0.84)	0.77 (0.72, 0.82)	0.77 (0.72, 0.81)	0.78 (0.74, 0.83)	0.60 (0.55, 0.65)
AUPRC[c]	0.25 (0.18, 0.33)	0.22 (0.16, 0.30)	0.23 (0.16, 0.30)	0.22 (0.15, 0.30)	0.06 (0.05, 0.08)
Sensitivity	0.70 (0.62, 0.82)	0.71 (0.62, 0.78)	0.72 (0.57, 0.83)	0.70 (0.62, 0.85)	0.63 (0.48, 0.78)
Specificity	0.78 (0.66, 0.83)	0.75 (0.70, 0.85)	0.71 (0.62, 0.86)	0.76 (0.60, 0.82)	0.58 (0.44, 0.72)
Balanced accuracy	0.74 (0.71, 0.77)	0.73 (0.70, 0.76)	0.72 (0.69, 0.75)	0.73 (0.70, 0.77)	0.60 (0.58, 0.65)
Precision	0.13 (0.10, 0.16)	0.12 (0.10, 0.15)	0.11 (0.09, 0.16)	0.12 (0.09, 0.16)	0.07 (0.06, 0.08)
F1 score	0.22 (0.17, 0.26)	0.20 (0.17, 0.25)	0.18 (0.15, 0.26)	0.21 (0.16, 0.25)	0.12 (0.10, 0.15)
PLR[d]	3.10 (2.25, 4.29)	2.84 (2.35, 4.38)	2.49 (2.02, 4.46)	2.90 (2.04, 3.91)	0.64 (0.44, 0.77)
NLR[e]	0.39 (0.24, 0.49)	0.39 (0.28, 0.50)	0.39 (0.25, 0.53)	0.40 (0.21, 0.49)	1.49 (1.29, 1.92)

OSR					
**Measure[a]**	**RandomForest**	**LASSO**	**Elastic Net**	**Ridge**	**SVM**
AUC[b]	0.88 (0.85, 0.91)	0.86 (0.82, 0.89)	0.87 (0.83, 0.90)	0.87 (0.84, 0.90)	0.66 (0.60, 0.71)
AUPRC[c]	0.39 (0.30, 0.47)	0.33 (0.25, 0.42)	0.33 (0.25, 0.42)	0.34 (0.27, 0.43)	0.14 (0.09, 0.20)
Sensitivity	0.81 (0.76, 0.89)	0.74 (0.70, 0.83)	0.74 (0.70, 0.84)	0.84 (0.73, 0.89)	0.40 (0.34, 0.77)
Specificity	0.81 (0.75, 0.83)	0.85 (0.80, 0.86)	0.86 (0.79, 0.87)	0.76 (0.75, 0.88)	0.87 (0.49, 0.90)
Balanced accuracy	0.81 (0.78, 0.84)	0.80 (0.77, 0.83)	0.80 (0.78, 0.84)	0.80 (0.78, 0.84)	0.63 (0.61, 0.68)
Precision	0.17 (0.13, 0.20)	0.19 (0.15, 0.22)	0.20 (0.15, 0.23)	0.14 (0.13, 0.23)	0.13 (0.07, 0.17)
F1 score	0.28 (0.23, 0.32)	0.30 (0.25, 0.34)	0.31 (0.25, 0.35)	0.24 (0.22, 0.35)	0.19 (0.12, 0.24)
PLR[d]	4.22 (2.92, 4.94)	4.88 (3.78, 5.79)	5.12 (3.50, 6.05)	3.47 (3.30, 6.36)	0.69 (0.42, 0.76)
NLR[e]	0.23 (0.12, 0.31)	0.30 (0.19, 0.37)	0.30 (0.18, 0.37)	0.22 (0.13, 0.34)	3.10 (1.46, 4.43)

Lasso, least absolute shrinkage and selection operator; OSO, order status only; OSR, order status and result.

[a]Calculations were completed with the validation set, and 95% CIs were computed using 2000 bootstrap replicates for each performance measure.

[b]AUC: area under the receiver operating characteristic curve.

[c]AUPRC: area under the precision recall curve.

[d]PLR: positive likelihood ratio.

[e]NLR: negative likelihood ratio.
