# Supplementary material for: Predicting Adverse Outcomes for Febrile Patients in the Emergency Department Using Sparse Laboratory Data: Development of a Time Adaptive Model
Source: JMIR Med Inform. 2020 Mar 26;8(3):e16117. doi: 10.2196/16117 (PMC7146241; doi:10.2196/16117)
Supplement: Multimedia Appendix 4 [file medinform_v8i3e16117_app4.pdf]

Multimedia Appendix 4. Receiver operating characteristic (ROC) curves for models.

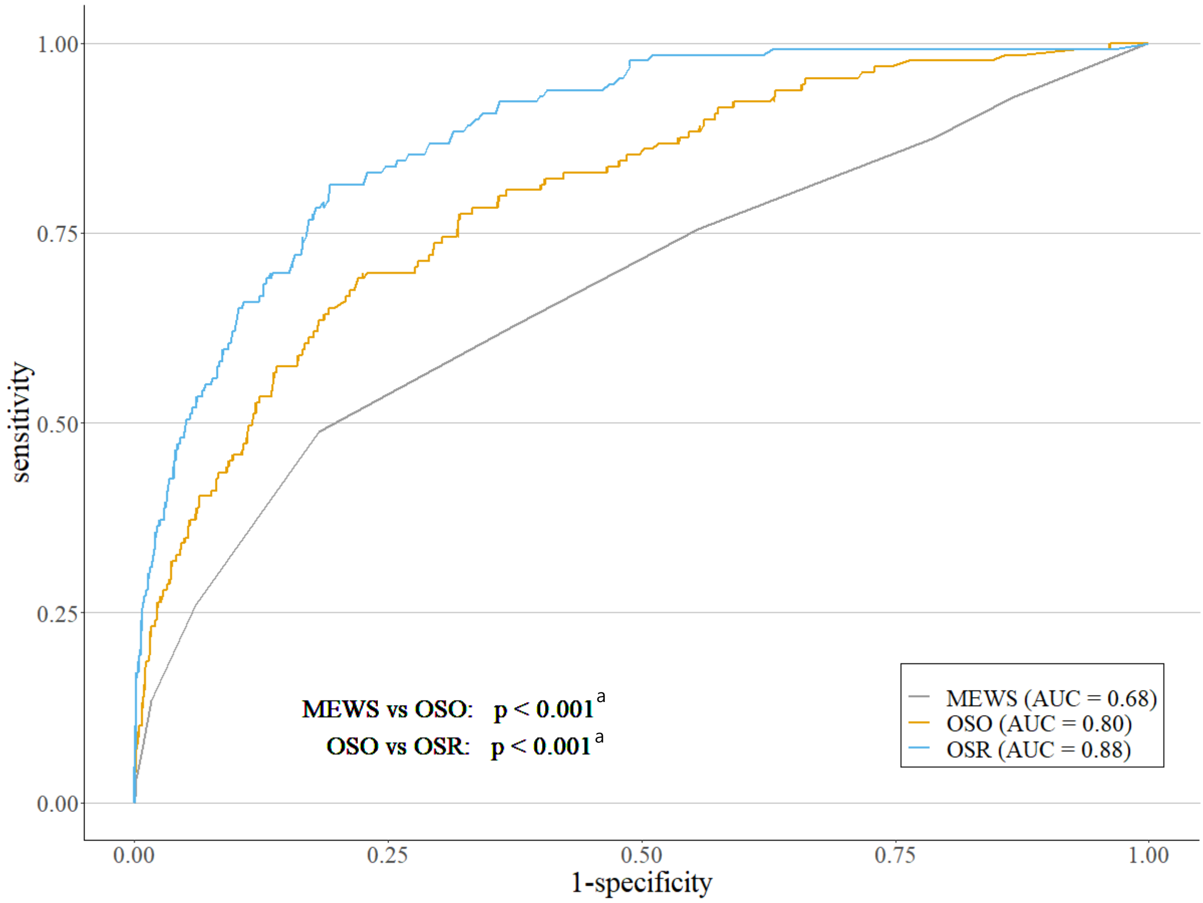

[a] Comparison of ROC curves of the two models using DeLong's test.
