# Supplementary material for: Predicting Adverse Outcomes for Febrile Patients in the Emergency Department Using Sparse Laboratory Data: Development of a Time Adaptive Model
Source: JMIR Med Inform. 2020 Mar 26;8(3):e16117. doi: 10.2196/16117 (PMC7146241; doi:10.2196/16117)
Supplement: Multimedia Appendix 5 [file medinform_v8i3e16117_app5.pdf]

**Multimedia Appendix 5.** Important variables selected from developed models.

The following two scatter plots and tables were drawn to examine the relationship between odds ratios and important variables from random forest and elastic net in the OSO and OSR models. There is consistency in the important tests from both models, and the correlation is higher in the OSR model where information is added than that in the OSO model.

Plots of important variables from the OSO and the OSR model

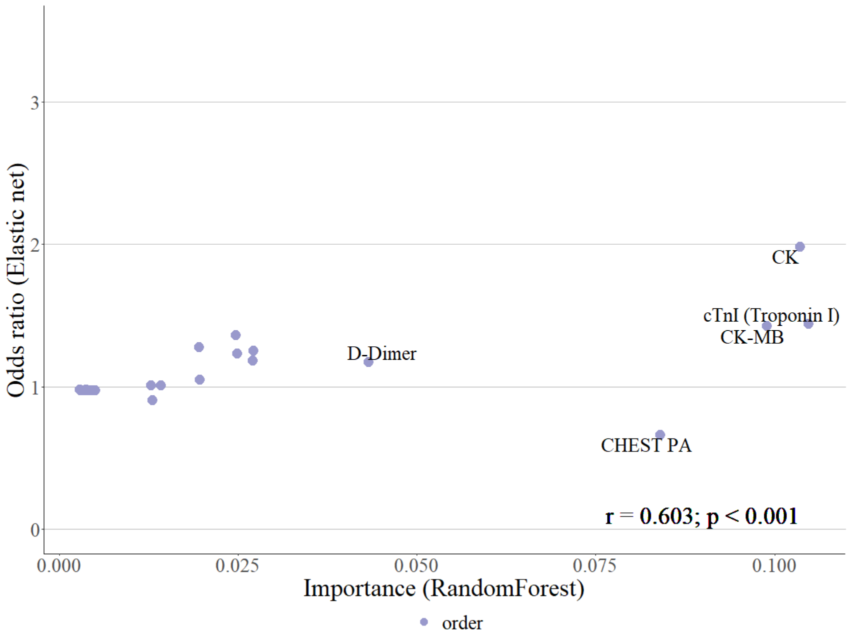


CK, creatine kinase; cTnl (Troponin I), cardiac troponin I; CK-MB, creatine kinase-MB

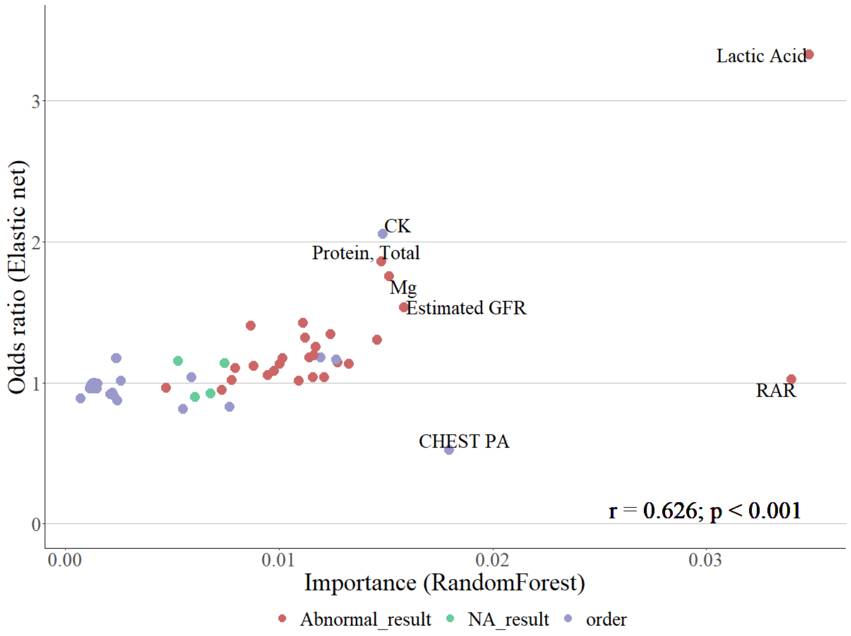


CK, creatine kinase; Mg, magnesium; RAR; the number of rarely detected abnormal results

Tables of important variables from the OSO and the OSR model

| Rank | OSO | | | |
|---|---|---|---|---|
| | RandomForest | | Elastic Net | |
| | **Laboratory Test** | **Importance** | **Laboratory Test** | **Odds ratio** |
| 1 | Cardiac troponin I (cTnI (Troponin I)) | 0.10 | Creatine kinase (CK) | 1.99 |
| 2 | Creatine kinase (CK) | 0.10 | Cardiac troponin I (cTnI (Troponin I)) | 1.44 |
| 3 | Creatine kinase-MB (CK-MB) | 0.10 | Creatine kinase-MB (CK-MB) | 1.43 |
| 4 | CHEST PA | 0.08 | Alkaline Phosphatase (ALP) | 1.36 |
| 5 | D-Dimer | 0.04 | Antibody Screen | 1.28 |
| 6 | The number of rarely ordered tests (ROT) | 0.04 | Calcium | 1.25 |
| 7 | Calcium | 0.03 | Ionized calcium | 1.23 |
| 8 | Osmolality, Serum | 0.03 | Osmolality, Serum | 1.18 |
| 9 | Ionized calcium | 0.02 | D-Dimer | 1.17 |
| 10 | Alkaline Phosphatase (ALP) | 0.02 | GGT (Gamma-Glutamyl Transferase) | 1.04 |

| Rank | OSR | | | | | |
|---|---|---|---|---|---|---|
| | RandomForest | | | Elastic Net | | |
| | **Laboratory Test** | **Type** | **Impor -tance** | **Laboratory Test** | **Type** | **Odds ratio** |
| 1 | Lactic Acid | Result (abnormality) | 0.03 | Lactic Acid | Result (abnormality) | 3.32 |
| 2 | The number of rarely detected abnormal results (RAR) | Result (abnormality) | 0.03 | Creatine kinase (CK) | Order | 2.05 |
| 3 | CHEST PA | Order | 0.02 | Protein, Total | Result (abnormality) | 1.86 |
| 4 | Estimated GFR | Result (abnormality) | 0.02 | Magnesium (Mg) | Result (abnormality) | 1.75 |
| 5 | The number of rarely ordered tests (ROT) | Order | 0.02 | Estimated GFR | Result (abnormality) | 1.54 |
| 6 | Magnesium (Mg) | Result (abnormality) | 0.02 | Prothrombin Time (International Normalized Ratio) | Result (abnormality) | 1.42 |
| 7 | Creatine kinase (CK) | Order | 0.01 | D-Dimer | Result (abnormality) | 1.40 |
| 8 | Protein, Total | Result (abnormality) | 0.01 | Bilirubin, Total | Result (abnormality) | 1.34 |
| 9 | Blood Urea Nitrogen | Result (abnormality) | 0.01 | Phosphate | Result (abnormality) | 1.32 |
| 10 | Albumin, Blood | Result (abnormality) | 0.01 | Blood Urea Nitrogen | Result (abnormality) | 1.30 |
