# Supplementary material for: Predicting Adverse Outcomes for Febrile Patients in the Emergency Department Using Sparse Laboratory Data: Development of a Time Adaptive Model
Source: JMIR Med Inform. 2020 Mar 26;8(3):e16117. doi: 10.2196/16117 (PMC7146241; doi:10.2196/16117)
Supplement: Multimedia Appendix 6 [file medinform_v8i3e16117_app6.pdf]

Multimedia Appendix 6. Model performance without and with applying imbalance-easing techniques

	OSO		
**Measure[a]**	**Raw[b]**	**ADASYN (1:8)[c]**	**Random oversampling(1:9)[c]**
AUC[d]	0.769 (0.724, 0.813)	0.775 (0.73, 0.817)	0.772 (0.73, 0.821)
AUPRC[e]	0.226 (0.16, 0.3)	0.232 (0.142, 0.291)	0.239 (0.147, 0.314)
Sensitivity	0.721 (0.574, 0.833)	0.612 (0.6, 0.856)	0.767 (0.612, 0.84)
Specificity	0.71 (0.615, 0.86)	0.797 (0.585, 0.831)	0.678 (0.618, 0.829)
Balanced accuracy	0.716 (0.69, 0.75)	0.705 (0.694, 0.757)	0.723 (0.696, 0.761)
Precision	0.106 (0.085, 0.164)	0.125 (0.083, 0.163)	0.102 (0.087, 0.154)
F1 score	0.184 (0.153, 0.258)	0.208 (0.151, 0.26)	0.179 (0.156, 0.249)
NLR[f]	0.393 (0.249, 0.533)	0.486 (0.206, 0.52)	0.343 (0.219, 0.496)
PLR[g]	2.489 (2.016, 4.458)	3.02 (1.941, 4.624)	2.38 (2.02, 3.859)

[a]Calculations were completed with the validation set, and 95% CIs were computed using 100 bootstrap replicates for each performance measure.

[b]Raw: the performance from the OSO model developed by elastic net algorithms using original imbalanced data.

[c]Ratio: the ratio between the minority and majority classes.

[d]AUC: area under the receiver operating characteristic curve.

[e]AUPRC: area under the precision recall curve.

[f]NLR: negative positive likelihood ratio.

[g]PLR: positive likelihood ratio.

OSR			
**Measure[a]**	**Raw[b]**	**ADASYN (1:7)[c]**	**Random oversampling(1:9)[c]**
AUC[d]	0.868<br>(0.834, 0.898)	0.868<br>(0.818, 0.894)	0.859<br>(0.834, 0.901)
AUPRC[e]	0.332<br>(0.254, 0.422)	0.343<br>(0.218, 0.423)	0.335<br>(0.245, 0.438)
Sensitivity	0.744<br>(0.701, 0.841)	0.69<br>(0.648, 0.857)	0.783<br>(0.714, 0.871)
Specificity	0.855<br>(0.785, 0.869)	0.893<br>(0.714, 0.898)	0.806<br>(0.744, 0.865)
Balanced accuracy	0.799<br>(0.775, 0.837)	0.792<br>(0.755, 0.824)	0.795<br>(0.773, 0.835)
Precision	0.196<br>(0.148, 0.226)	0.235<br>(0.119, 0.255)	0.161<br>(0.13, 0.221)
F1 score	0.31<br>(0.248, 0.351)	0.35<br>(0.208, 0.379)	0.267<br>(0.225, 0.343)
NLR[f]	0.299<br>(0.181, 0.366)	0.347<br>(0.178, 0.422)	0.269<br>(0.153, 0.35)
PLR[g]	5.119<br>(3.498, 6.049)	6.464<br>(2.546, 7.233)	4.044<br>(3.116, 5.966)

[a]Calculations were completed with the validation set, and 95% CIs were computed using 100 bootstrap replicates for each performance measure.

[b]Raw: the performance from the OSO model developed by elastic net algorithms using original imbalanced data.

[c]Ratio: the ratio between the minority and majority classes.

[d]AUC: area under the receiver operating characteristic curve.

[e]AUPRC: area under the precision recall curve.

[f]NLR: negative positive likelihood ratio.

[g]PLR: positive likelihood ratio.
